# On the Legal Responsibility of Pregnant Women

**Published:** 1863-10

**Authors:** William Sedgwick


### Art. IX.—ON THE LEGAL RESPONSIBILITY OF PREGNANT WOMEN.

By William Sedgwick, M.R.C.S. and L.S.A.

The comparative rarity with which cases involving the question of the mental condition of women during pregnancy comes before the notice of our criminal courts will be a sufficient motive for placing on record the following case of murder of an only child, aged two years and three months, and attempted suicide, by a young woman in the fourth month of her third pregnancy, which has lately occurred in the parish of Marylebone, and has been followed by her trial and acquittal on the ground of insanity consequent on pregnancy. The case has acquired an additional interest from having been followed, after an interval of only a few weeks, by a parallel case of murder of an only child, of about the same age, and attempted suicide, by a young woman during the same period of her pregnancy, and residing in a house very near that in which the first murder was committed.

On June 10, 1863, at 8·20 P.M., I was sent for to attend Mary Ann Payne, a married woman, aged 21 years, who had thrown herself from a back bed-room window on the second floor, and whom, on my arrival at the house, I found lying in her night-dress on a sofa in the front parlour, in a state of complete collapse. She had received severe injuries on both legs, but no bones were broken. There were several bruises on both arms, and two or three on the head. An interrupted scratch extended about four inches round the neck, showing that a very feeble attempt at cutting her throat had been made with a blunt knife. The insensibility continued for about eight minutes after my arrival, and when she had revived, the only information that could at first be obtained from her was that she had slipped off the door-step and fainted. She appeared to have no recollection of having fallen or thrown herself from a window; and it was not known that any murder had been committed until I sent one of the women in attendance upstairs for a blanket, who, on turning down the bedclothes, found the body of a child, with its throat cut completely across. On being told of the discovery, I immediately went up to the room and ascertained that the body of the child was still warm, but that the head and extremities were cold. The wound in the throat had a jagged appearance, as if made with a blunt knife, and great force must have been used, as all the intervening structures had been cut across down to the spine, which was laid bare, so that when the body was raised the head fell back between the shoulder blades. On the floor near the bed, a constable, who had been sent for, picked up a common table-knife with a very blunt edge, which had been smeared with blood and wiped; a piece of paper folded in two and stained with blood, which had evidently been used for wiping the knife, was found under the bed. There was a basin on the table containing a large quantity of vomit, which was subsequently found, on analysis, to contain opium; and a small remainder of laudanum was found in a six-ounce bottle and in a wine-glass in the same room. The window was wide open, finger-marks of blood were observed on the window-sill, and a small flowerpot outside, which had apparently stood in the way, was overturned. On going down to the yard beneath the window, patches of blood in three places were observed on the flagstones, and the two ends of a clothes-line, which had previously been stretched across the yard with a quilt and other things on it, were noticed hanging against the side-walls, the line having been broken by the woman in falling from the window, and having so probably saved her life. By the following morning she had sufficiently recovered to appear at the Marylebone Police Court, where she was committed for trial on the charge of wilful murder, and was tried at the Central Criminal Court on July 14, 1863, when the jury almost immediately returned a verdict of Not Guilty, upon the ground of insanity, and the prisoner was ordered to be confined during her Majesty's pleasure.

It was proved in evidence at the trial that she was four months advanced in her third pregnancy, and that she had for some weeks previously been in a very desponding state of mind; that this despondency had been characteristic of her former pregnancies, and that, on several occasions, during the present and the previous pregnancies, she had expressed a dread of her husband's razors, and likewise of common knives being left within her reach. In the intervals between her pregnancies she had always appeared rational. The only family history of insanity obtained was that one of her great aunts, who was the sister of her paternal grandmother, had committed suicide by cutting her throat, leaving a husband and three young children. There was conclusive evidence to establish that the prisoner had always shown great affection for the murdered boy, who was her only child, the second pregnancy having resulted in a miscarriage; that on the afternoon of the day when the murder was committed, she had taken him out with her for a walk, and that during her walk she had called on three chemists, from each of whom she had purchased laudanum, amounting in all to five pennyworth; that on her return home she had left the child on the ground-floor to play with her landlady's children, and had gone upstairs alone to her own room, where she remained till tea-time; that she then went downstairs for the child, and took him with her to their room, for the purpose, it was supposed, of having tea; after which, it appears from her own statement, that the child was undressed and put to bed, and that, feeling unwell, she also undressed and went to bed, lying down by the side of the child. No clear account could be obtained of the subsequent proceedings, as the prisoner professed to have lost almost all recollection of what occurred afterwards; but from the few imperfect statements obtained from her, and from the collateral evidence adduced at the trial, it is probable that she commenced the tragedy by swallowing the laudanum, which appears to have been almost immediately rejected with the contents of a full stomach, consisting of a very hearty and almost undigested dinner of meat and vegetables, for she never exhibited any symptoms of poisoning by opium. At what time she made the attempt to cut her own throat is quite uncertain, for it may have immediately preceded or followed the cutting of her child's throat. The murder of the child may, with great probability, be inferred to have occurred about 8 p.m., at which time her husband was accustomed to return home from his work as an engineer, and she was enabled to tell the exact moment of his arrival in the house by the ringing of a signal-bell over the street-door when he let himself in, for it was proved that he had scarcely had time to reach the first landing on the staircase before she had thrown herself from the window, and he was called back by the cries of the landlady in the back parlour, who had seen her fall, and who hastened to pick her up; and, consequently, as the body of the child was still warm when I saw it, about 8.30 p.m., it is evident that the murder must have almost immediately preceded the last of her three attempts at suicide.

The counsel's defence of the prisoner was based, almost exclusively, on the fact that women during pregnancy have sometimes been observed to become more or less insane, and that, as the evidence adduced at the trial proved that the prisoner had suffered from melancholy, amounting at times to unsoundness of mind, during three successive pregnancies, and had committed murder and attempted suicide without any apparent motive, she was therefore entitled to a verdict of not guilty, on the ground of insanity. No special evidence was adduced to prove the occurrence of insanity in other cases during pregnancy; no recorded case of the same or of a corresponding character was cited, and there was no mention at the trial of any hereditary influence in this case; for the fact of the paternal grandmother's sister having committed suicide, although interesting in a medical point of view, would probably have been considered to indicate too slight and remote a relationship to have affected the decision of the jury. In the summing up of the case, the judge (Mr. Justice Wightman) dwelt chiefly on the facts adduced in favour of insanity, and no further evidence seemed to be expected or required to prove that women are liable to become insane in consequence of pregnancy, and that, under such circumstances, they are not legally responsible for their acts.

In addition to the above well-marked illustration of insanity developed during pregnancy, I am enabled, from private sources of information, to refer to three others in which pregnancy was associated with mental derangement. 1st. The case of a lady residing in the north district of London, who suffered from well-marked insanity during the early period of pregnancy, and which lasted for two weeks; she recovered completely, and did not have any return of insanity during her subsequent pregnancies. 2nd. The case of a woman, aged 26 years, the wife of a cabinet-maker and the mother of six children, who was quite insane during each of her six pregnancies, and was to a very slight extent insane during the short intervals between them: her mother was an inmate of St. Luke's Asylum, for incurable insanity. 3rd. The case of a married woman, which came under the observation of Mr. W. O. Chalk, in which epilepsy was suddenly developed in the fourth month of her pregnancy, and was followed by insanity, in consequence of which premature labour was, at a later period of her pregnancy, induced, and the patient recovered her reason.

From the recorded experience of medical authorities on insanity developed during pregnancy, the following evidence may be cited. Dr. James Reid, in his paper "On the Causes, Symptoms, and Treatment of Puerperal Insanity,"* states that "the cases of insanity which arise *during* pregnancy are much smaller in number than those which *follow* it, and the majority of the former terminate with the occurrence of labour." He has recorded two cases which were admitted into the Bethnal Green Asylum, and it may be useful to quote the first, as it resembles very closely the case which has lately been tried at the Central Criminal Court: it is that of a female "who had been attacked by melancholia immediately after quickening, with a strong desire to destroy herself and her three children; it continued during the remainder of pregnancy, and became worse after delivery." —Dr. Montgomery† "noticed a case where mania occurred in eight successive pregnancies; and another in which the woman was three times similarly affected soon after conception, and remained deranged until within a short time of her labour, when she became sane, and continued so until her next pregnancy." A somewhat similar case to this last, as regards the early development of insanity during pregnancy, is recorded by Esquirol,‡ of a young woman of a sensitive habit, who had an attack of madness in two successive pregnancies, commencing immediately after conception, and lasting fifteen days. Dr. Churchill§ relates the following case: "A lady pregnant, but in perfect health, was employed in some household duty, and was talking cheerfully to her husband and sister. Suddenly, and without any apparent reason, she left them, went to her bed-room, and in-

* *Journal of Psychological Medicine*, vol. 1, p. 132. 1848.

† *An Exposition of the Signs and Symptoms of Pregnancy*, 2nd edit. p. 36. 1856.

‡ Dr. Burrows: *Commentaries on Insanity*. p. 464.

§ *On the Diseases of Women, including those of Pregnancy and Child-bed*, 4th edit. p. 478–9. 1857.

stantly destroyed herself. This (adds Dr. Churchill) must have resulted from a sudden attack of insanity, but up to the moment before she was cheerful and happy, in good circumstances, and greatly attached to her husband; but other members of her family have been subject to insanity."—Dr. Tapson* has recorded the case of a married woman, aged 31 years, who was admitted into the North London Hospital, July 6, 1842. She had given birth to four children, two of whom had died, and the youngest surviving child was five weeks old. Her father, who had died some years previously, was insane for four months about two years previous to his death; he recovered his reason, but died from hemiplegia. On the mother's side, two cousins were, at the time of the patient's admission, in lunatic asylums. It appears that, about the fourth month of her last pregnancy, she was suddenly attacked with melancholia, after a violent quarrel with another woman, and that since that time she has "always felt distracted." She had a great desire to commit suicide, and the love which she had previously felt for her elder child was so changed to hatred, that she probably would, if unrestrained, have murdered it. Dr. Burrows† remarks: "Some (women) are insane on every pregnancy or lying-in, others only occasionally;" and he refers to two cases which had come under his own observation, "where hysterical symptoms attended during pregnancy, and the patients, almost immediately on delivery, became insane." Dr. Lever,‡ Dr. Copland,§ and many other writers on the subject, bear testimony to the influence of pregnancy in developing insanity, especially in those females who are hereditarily predisposed to the disease.—Lastly, Dr. L. V. Marcé ‖ published, in 1858, a special treatise on this subject, in which he has collected nineteen cases of insanity occurring in pregnant women, in sixteen of which the form of mental alienation was exactly noted: of these, ten presented the condition of mental depression; and "in three patients ideas of suicide were predominant, one of whom, under the influence of delirious ideas, formed even projects of murder against her child." In one of the patients referred to by Dr. Marcé, the insanity occurred at the end of seven successive pregnancies; and in another patient, the mother of ten children, the melancholy symptoms showed themselves during the first months of her ten pregnancies, and disappeared habitually with

---

* Fellowes' Clinical Prize Reports, *Medical Gazette*, June 9, 1843, pp. 392-4.

† *Commentaries on the Causes, Forms, Symptoms, and Treatment of Insanity*, p. 364. 1828.

‡ *Guy's Hospital Reports*, 2nd series, vol. v. p. 22.

§ *Dictionary of Practical Medicine*, Art. "Puerperal Insanity," vol. ii. p. 543.

‖ *Traité de la Folie des Femmes Enceintes, des Nouvelles Accouchées et des Nourrices, et des Considérations Médico-legales qui se rattachent à ce sujet.*

delivery; two years after the birth of her last child this latter patient became quite mad.

The nature and extent of this morbidly sympathetic influence of the uterus on the mind are sometimes very forcibly illustrated in cases of uterine disturbance unaccompanied by pregnancy, as in the following case* of a lady, who during her first pregnancy was attacked with mental alienation; ten years afterwards, owing to the mental disorder being renewed, it was thought that she was again pregnant. Boyer, the celebrated French accoucheur, was consulted, and recognised an uterine polypus, the removal of which put an end to the mental derangement. In like manner also suppression of the menses, independent of pregnancy, may occasionally react on the brain and cause transient insanity. Dr. Rose Cormack† relates two cases of transient insanity from this cause, one observed by himself and one by Dr. Merrem; and in the somewhat celebrated case of kleptomania tried at the Cumberland Midsummer Sessions, Carlisle, July 1, 1845, the prisoner was "acquitted on the ground of temporary insanity from suppression of the menses."‡

From the preceding observations it may be inferred that insanity is occasionally liable to occur in pregnant women as a result of their pregnancy, especially in those who are hereditarily predisposed to the disease; and from this an important inquiry in medical jurisprudence results, respecting the extent to which the condition of pregnancy itself, independent of legal proof of insanity, can justly be urged as an excuse for crime. Dr. Taylor§ informs us that "females in the *pregnant* state have been known to perpetrate murder apparently from some sudden perversion of their moral feelings: there has no doubt been latent intellectual disturbance, but not sufficient to attract the notice of friends. I am not aware (adds Dr. Taylor) that a plea of exculpation on the ground of insanity has been admitted in this country under these circumstances." Whilst Devergie‖ is of opinion that pregnancy does not of itself so disturb the intellectual faculties as to render a woman incapable of self-control, on the other side, Dr. Jörg¶ has written a special work on the legal responsibility of women during pregnancy and parturition, in which he advocates the doctrine of their non-responsibility as regards injuries inflicted on themselves or others. On a ques-

---

* Quoted by Marcé, op. cit. p. 13.

† "On Transient Insanity, chiefly in reference to Forensic Medicine: with Cases." (*Monthly Journal of Medical Science*, vol. iii. pp. 903–913. 1843.)

‡ *Monthly Journal of Medical Science*, vol. v. pp. 632–6. 1845.

§ *Medical Jurisprudence*, 7th edit. p. 909. 1861.

‖ *Médecine Légale*, tom. i. p. 433.

¶ *Die Zurechnungsfähigkeit der Schwangern und Gebärenden beleuchtet.*

tion involving such important interests, and about which such conflicting opinions have been entertained, it is not to be expected that any clearly defined expression of opinion can be offered, applicable to all the offences committed by women during pregnancy; for however strongly marked in many cases the influence of pregnancy may appear to be on the mind, it would neither be safe nor right to admit in its full extent the doctrine of their legal irresponsibility for crime which has been advocated by Dr. Jörg, and which is apparently based on the erroneous opinion that pregnancy is a morbid, instead of being (as Dr. Denman very justly described it) simply an altered state of the system. Little or no assistance in solving this difficulty can be derived from the statistical reports of asylums for the insane, owing to the fact that the insanity developed during pregnancy is usually transient, or at least ceases when the child is born; and such cases are consequently subject, as a rule, to the care and protection of private friends, whose watchfulness serves to check the commission of any grave offence, and to restrain the patient from attracting public notice. But whilst the absence of notoriety is to be remarked in the more severe cases of mental disturbance during pregnancy, it is a subject of common observation that, in less severe cases, eccentricities of conduct and minor offences often come under public notice, and that women are by general consent absolved on the plea that they result from pregnancy, and are limited and peculiar to that condition; the power of self-restraint being, it is urged, so far weakened as to render women, at such times, as incapable of controlling their actions as they are of controlling those morbid fancies and desires which not unfrequently characterize pregnancy, and which, through their influence on the maternal system, are supposed to affect the development of the offspring. It is not therefore to be expected that cases of a very grave character, involving the legal responsibility of pregnant women, would, except on rare occasions, be brought under the notice of our criminal courts; and although I am not aware of the extent to which the passage quoted from Dr. Taylor may be admitted to prove that the case now recorded by myself is, as regards our own criminal literature, unique, yet even in foreign literature on medical jurisprudence the number of such cases is extremely small, so that I am only able to cite the following case, which came before the notice of one of the French criminal courts,* as being in any way parallel with that which has lately been decided at the Old Bailey; the most important difference be-

* *Annales d'Hygiène Publ.*, 1 ser. t. 17, p. 400] 1837 Quoted by Marcé, op. cit. pp. 132–3.

tween the two cases being the absence of any legal evidence of hereditary transmission of insanity in the one, and the strongly marked legal evidence of such transmission in the other. The case referred to is that of a woman who, during pregnancy, had inflicted mortal wounds on two of her children. It was proved that the mother and many of the relations of this woman had been mad. Leuret, the medical authority consulted at the trial, declared that taking into consideration the hereditary antecedents of this woman, her nervous temperament, her violent and excitable character, rendered still more irritable by the state of pregnancy, that it was not impossible that she had acted in consequence of some affection having momentarily disturbed the exercise of her mental faculties. The Court, taking into account this opinion, decided that the accused was guilty of the blows and wounds, but without the intention of causing death. In conclusion, it may be useful to contrast the ready acceptance, in our own time, of the plea of insanity in such cases, and the consequent acquittal of the prisoner, with the frequency with which, in former times, females in the pregnant state were found guilty of murder and other capital offences, and condemned to death, without any such plea in their favour being urged. And although there seems to be a tendency in modern times to abuse this plea of insanity in some of our late trials for murder, still, as regards the special class of cases referred to in this paper, in which the offence has been committed by women during pregnancy, it can hardly be doubted that our criminal law in old times was unjustly severe, not simply in omitting to temper justice with mercy, but in excluding, as was then the custom, this extenuating circumstance; and hence it may be inferred that many women have suffered death wrongfully, because the plea of insanity consequent on pregnancy was not urged in their favour.

---

